# Impaired Mitophagy of Nucleated Erythroid Cells Leads to Anemia in Patients with Myelodysplastic Syndromes

**DOI:** 10.1155/2018/6328051

**Published:** 2018-06-03

**Authors:** Huijuan Jiang, Liyan Yang, Lifang Guo, Ningbo Cui, Gaochao Zhang, Chunyan Liu, Limin Xing, Zonghong Shao, Huaquan Wang

**Affiliations:** Department of Hematology, General Hospital, Tianjin Medical University, Tianjin, China

## Abstract

Myelodysplastic syndromes (MDS) are a heterogeneous group of clonal stem cell disorders characterized by cytopenia and dysplasia. Anemia is the most common symptom in patients with MDS. Mitophagy and mitochondrial dysfunction might be involved in the development of MDS. In this study, we investigated the change of mitophagy in erythroid precursors in MDS patients. We found that NIX-mediated mitophagy was impaired in bone marrow nucleated red blood cells (NRBC) of MDS patients, associated with an increased amount of damaged mitochondria and increased ROS level which might lead to apoptosis and ineffective erythropoiesis. The results showed that the amount of mitochondria in GlycoA^+^ NRBC positively correlated with the count of ring sideroblasts in bone marrow samples. Meanwhile, the level of autophagy-associated marker LC3B in GlycoA^+^ NRBC had a positive correlation with hemoglobin (Hb) levels, and the amount of mitochondria in GlycoA^+^ NRBC had a negative correlation with Hb levels in high-risk MDS patients. Our results indicated that mitophagy might involve the pathogenesis of anemia associated with MDS. Autophagy might be a novel target in treatments of MDS patients.

## 1. Introduction

Myelodysplastic syndromes (MDS) are a heterogeneous group of clonal stem cell disorders characterized by ineffective and dysplastic hematopoiesis [1]. The most common symptom of MDS patients is anemia [2]. There are multiple potential mechanisms in anemia associated with MDS, such as poor response of erythropoietin (EPO) [3], altered GDF11-mediated Smad2/3 signaling [4, 5], and gene mutation [6]. Recently, some researchers have reported that mitochondrial DNA mutant mice could develop macrocytic anemia as a MDS-like phenotype [7]. Mitochondrial dysfunction could also lead to erythroid dysplasia or megaloblastic anemia [8].

Mitophagy, which occurs to defective mitochondria following damage or stress, is selective mitochondria degradation by autophagy. By eliminating and degrading depolarized mitochondria, mitophagy plays a critical role in maintaining healthy pools of mitochondria [9, 10]. There exists a functional relationship between mitophagy and differentiation of hematopoietic stem cells (HSC) [11]. Thus, impaired mitophagy might contribute to the development of MDS.

In this study, we investigated the change of mitophagy and mitophagy-associated markers in erythroid precursors of MDS patients to explore the correlation between impaired mitophagy and the pathogenesis of anemia associated with MDS.

## 2. Material and Methods

### 2.1. Patient Characteristics

A total of 54 patients with MDS newly diagnosed in the Department of Hematology of the General Hospital of Tianjin Medical University, Tianjin, China, were enrolled in this study from July 2015 to July 2016. This study included 30 males and 24 females with a median age of 60.5 (range 27–79 years) (Table 1). These MDS cases were classified according to the World Health Organization (WHO 2008) classification of myeloid neoplasms and acute leukemia: refractory anemia (RA: *n* = 2), refractory neutropenia (RN: *n* = 1), RA with ring sideroblasts (RARS: *n* = 5), refractory cytopenia with multiple dysplasia (RCMD: *n* = 15), RA with excess blasts type 1 (RAEB-1: *n* = 7), RAEB-2 (*n* = 23), and del (5q) syndrome (*n* = 1). Based on the International Prognostic Scoring System (IPSS) for MDS, there are four groups: the low-risk group (*n* = 3), intermediate-risk-1 group (INT-1: *n* = 25); intermediate-risk-2 group (INT-2: *n* = 17), and high-risk group (*n* = 9). The MDS patients were divided into two groups: the low-risk group (low-risk and intermediate-risk-1 cases) and the high-risk group (intermediate-risk-2 and high-risk cases).

Thirty-three non-MDS cases without anemia (immune thrombocytopenia *n* = 9, idiopathic granulocytopenia *n* = 24) were selected as controls in this study, which included 13 males and 20 females with a median age of 52 (range 24–74 years).

This study was approved by the Ethics Committee of the General Hospital of Tianjin Medical University. Informed written consent was obtained from all patients and controls or their guardians in accordance with the Declaration of Helsinki.

### 2.2. Flow Cytometry (FCM)

Bone marrow samples were collected in heparin anticoagulant tubes from MDS patients and controls. Nucleated red blood cells (NRBC) were stained with FITC/APC/PE-anti-GlycoA (BD Biosciences, USA). To test intracellular endogenous NIX and microtubule-associated protein 1-light chain 3 (LC3B), GlycoA^+^ NRBC samples were incubated with rabbit anti-human NIX primary antibody (LSBio, USA) and rabbit anti-human-LC3B-PE antibody (Cell Signaling Technology, USA) for 15 minutes. After washing with PBS, the samples were incubated with mouse anti-rabbit secondary antibody conjugated to R-Phycoerythrin (BD Biosciences, USA) for 20 minutes. To stain mitochondria, GlycoA^+^ NRBC were labeled with 100 nM of MitoTracker Deep Red (MTDR, Life Technologies, USA) at 37°C for 30 minutes [12]. To analyze mitochondrial depolarization, GlycoA^+^ NRBC were stained with 200 *μ*M of JC1 (Life Technologies, USA) at 37°C for 30 min. To measure intracellular ROS levels, GlycoA^+^ NRBC were washed and resuspended in staining buffer and loaded with 5-(and-6)-chloromethyl-2′,7′-dichlorodihydrofluorescein diacetate, acetyl ester (H_2_DCFDA, Sigma, USA) in the dark for 30 min at 37°C and 5% CO_2_. Intracellular fluorescent products were measured immediately by FCM. More than 30,000 cells were acquired using a FACSCalibur flow cytometer (BD Biosciences, USA) and analyzed using CellQuest software version 3.1 software (BD Biosciences, USA).

### 2.3. Autophagosomes Observed in NRBC

**Figure 1:**
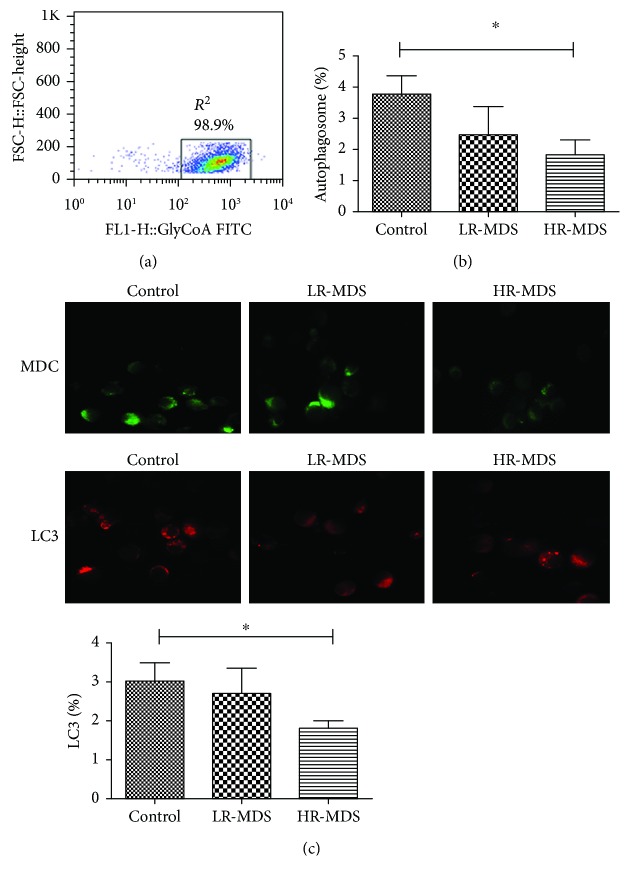
The autophagy level observed in NRBC (HR-MDS: high-risk MDS, LR-MDS: low-risk MDS). (a) The purity of sorted GlycoA^+^ NRBC tested by FCM (>95%). (b) The autophagosomes in GlycoA^+^ NRBC from high-risk MDS decreased compared with controls (^∗^ *P* < 0.05). (c) The LC3 expression in GlycoA^+^ NRBC from high-risk MDS was lower than that of controls (^∗^ *P* < 0.05).

After sorting by GlycoA microbeads (Bruker, Germany), the purity of sorted GlycoA^+^ NRBC was more than 95% (Figure 1(a)). Adjusting the cell density of 1 × 10^6^/ml, l ml of the cell suspension was incubated with 1 ml of monodansylcadaverine (MDC, 0.05 mmol/l) at 37°C and 5% CO_2_ for 45 min. After fixing by immunostaining for 10 min, the autophagosomes in green fluorescence was observed using a fluorescence microscope (Olympus, Japan). If there were more than 3 autophagosomes in the cells, then these were defined as autophagosome-positive cells. The total cell count was 200 cells and the percentage of autophagosome-positive cells was calculated. Every experiment was repeated at least 3 times.

### 2.4. Immunofluorescence

After fixing, rupturing of membranes, and blocking by 2% BSA, the sorted GlycoA^+^ NRBC smears were incubated with rabbit-anti-human LC3 polyclonal antibody (1 : 200 dilution, Cell Signaling Technology, USA) at 4°C overnight and washed with PBS 3 times (5 min each). Then, slips were incubated with PE-labeled mouse-anti-rabbit secondary antibody (1 : 500 dilution, Cell Signaling Technology, USA) at room temperature for 60 min and washed with PBS 3 times (10 min each). After the addition of 20 *μ*l of antifluorescence mounting media, the expression of LC3 protein in GlycoA^+^ NRBC was observed by a fluorescence microscope (Olympus, Japan). If there were more than 3 LC3 in the cells, then these were defined as LC3-positive cells. The total cell count was 200 cells and the percentage of LC3-positive cells was calculated. Every experiment was repeated at least 3 times.

### 2.5. Real-Time Quantitative Transcriptase-Polymerase Chain Reaction (Q-PCR)

GlycoA^+^ NRBCs were sorted by GlycoA microbeads (Bruker, Germany). Total RNA was extracted using TRIzol (Takara Bio USA Inc.), and cDNA was generated using a reverse transcriptase kit (Takara Bio USA Inc.). The gene expressions were quantified by Q-PCR (SYBR® Premix Ex Taq II, Takara Bio, China). The primer sequences were as follows: AMPK forward 5′-TTGAAACCTGAAAATGTCCTGCT-3′, reverse 5′-GGTGAGCCACAACTTGTTCTT-3′; ULK1 forward 5′-ACAGAGACCGTGGGCAAGT-3′ reverse 5′-CGACCTCCAAATCGTGCTT-3′; mTOR forward 5′-GCAGATTTGCCAACTACC-3′, reverse 5′-CACGGAGAACGAGGACA-3′; and GAPDH forward 5′-GCACCGTCAAGGCTGAGAAC-3′, reverse 5′-TGGTGAAGACGCCAGTGGA-3′. To generate the relative quantification (RQ) of the gene expression, the 2^−ΔΔCt^ method was used: ΔΔCt = (Ct_target_ − Ct_GAPDH_)_patients_ − (Ct_target_ − Ct_GAPDH_)_controls_.

### 2.6. Western Blot (WB)

The proteins of sorted GlycoA^+^ NRBC were extracted by lysis buffer (Biotech, Beijing, China). Protein concentration was determined using the Pierce BCA Protein Assay Kit (Thermo Scientific, USA). Samples were separated by 12% SDS-PAGE gels and then transferred to PVDF membranes (PE, USA) by a Trans-Blot Cell system (Bio-Rad, USA) using standard Western blotting procedures. The membranes were probed with a rabbit anti-human TOM20 antibody (Cell Signaling Technology, number 13929, USA) at 1 : 1000 and incubated overnight at 4°C. The rabbit anti-human *β*-actin antibody (Santa Cruz Biotechnology Inc., sc-47778, USA) at 1 : 5000 was used as loading control. The goat anti-rabbit IgG HRP-conjugated secondary antibody (Santa Cruz Biotechnology Inc., sc-2054, USA) was incubated for 1 hour at room temperature. After washing, an electrochemiluminescence (ECL) reagent (Thermo Scientific, number 32106, USA) was used for chemiluminescence.

### 2.7. Statistical Analysis

All data were analyzed using SPSS 21.0 (SPSS Inc., Chicago, IL, USA). Data were presented as mean ± SD. ANOVA analysis was used for three independent groups. Pearson correlation analysis was used for analyzing correlation, while Spearman correlation analysis was used for nonnormal distribution data. Statistical significance was defined as *P* < 0.05.

## 3. Results

### 3.1. The Autophagy in GlycoA^+^ NRBC Decreased in High-Risk MDS Patients

Autophagosomes in GlycoA^+^ NRBC observed by MDC in the high-risk MDS group (1.83 ± 0.46%) decreased compared with controls (3.78 ± 0.58%, *P* = 0.0392). There was no significant difference between the controls and the low-risk MDS group (2.78 ± 0.92%, *P* > 0.05) (Figure 1(b)). The LC3 expression in GlycoA^+^ NRBC from the high-risk MDS group (1.45 ± 0.32%) was lower than that of controls (3.03 ± 0.45%, *P* = 0.0456). There was no significant difference between the controls and the low-risk MDS group (2.68 ± 0.65%, *P* > 0.05) (Figure 1(c) and Supplementary Figure 1).

### 3.2. The Mitophagy in GlycoA^+^ NRBC Decreased in High-Risk MDS Patients

**Figure 2:**
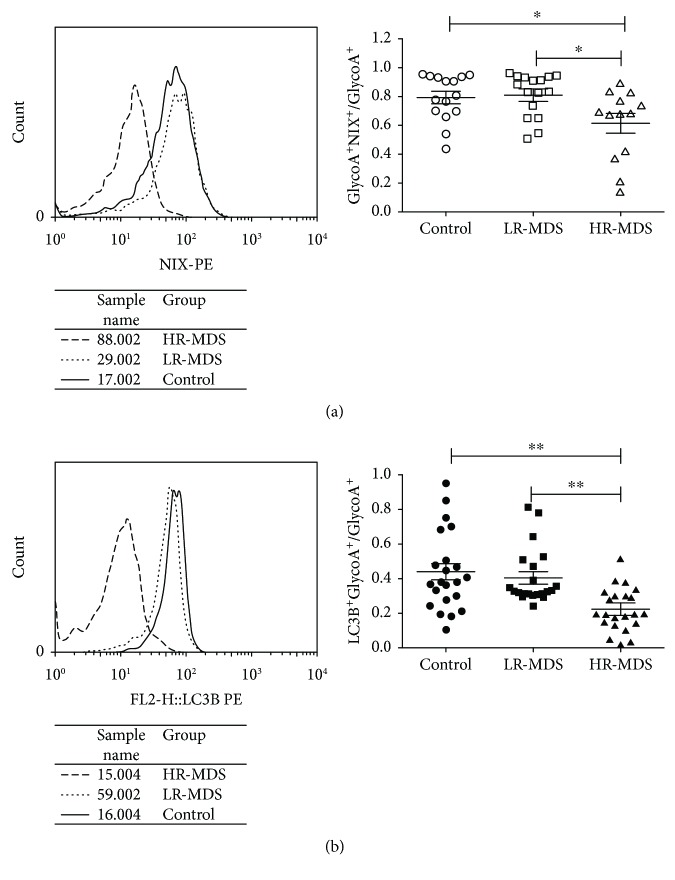
The expression of NIX and LC3B in GlycoA^+^ NRBC measured by FCM. (a)The level of NIX in GlycoA^+^ NRBC in high-risk MDS was lower than that of controls and low-risk MDS. (b) The level of LC3B in GlycoA^+^ NRBC in high-risk MDS was lower than that of controls and low-risk MDS (^∗^ *P* < 0.05, ^∗∗^ *P* < 0.01).

The expression of mitophagy receptor NIX and LC3B in GlycoA^+^ NRBC decreased in high-risk MDS patients. The expression of NIX in GlycoA^+^ NRBC in the high-risk group (*n* = 13, 0.61 ± 0.24) was lower than that in controls (*n* = 15, 0.79 ± 0.16, *P* = 0.0266) and the low-risk group (*n* = 16, 0.81 ± 0.15, *P* = 0.0112). There was no significant difference between the controls and the low-risk group (*P* = 0.7387) (Figure 2(a)). The expression of LC3B in GlycoA^+^ NRBC in high-risk MDS patients (*n* = 23, 0.22 ± 0.12) was lower than that in controls (*n* = 22, 0.43 ± 0.22, *P* = 0.0003) and low-risk MDS patients (*n* = 21, 0.40 ± 0.16, *P* = 0.0001). There was no significant difference between the controls and the low-risk group (*P* = 0.6218) (Figure 2(b)).

### 3.3. The mRNA Expression of Autophagy Regulator Gene Measured by Q-PCR

**Figure 3:**
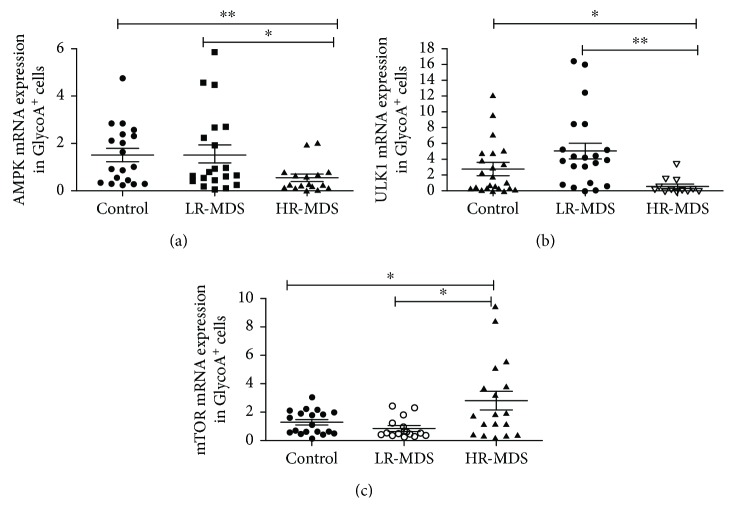
The mRNA expression of the autophagy regulator gene in GlycoA^+^ NRBC measured by Q-PCR. (a) The AMPK mRNA expression in GlycoA^+^ NRBC of high-risk MDS was lower than that in controls and low-risk MDS. (b) The ULK1 mRNA expression in GlycoA^+^ NRBC of high-risk MDS was lower than that in controls and low-risk MDS. (c) The mTOR mRNA expression in GlycoA^+^ NRBC of high-risk MDS was higher than that in controls and low-risk MDS (^∗^ *P* < 0.05, ^∗∗^ *P* < 0.01).

The mRNA expression of autophagy promotor genes—AMPK and ULK1—in GlycoA^+^ NRBC decreased in high-risk MDS patients. The expression of AMPK mRNA in GlycoA^+^ NRBC in the high-risk group (*n* = 17, 0.53 ± 0.61) was lower than that in controls (*n* = 19, 1.51 ± 1.25, *P* = 0.0062) and the low-risk group (*n* = 20, 1.55 ± 1.70, *P* = 0.0255). There was no significant difference between the controls and the low-risk group (*P* = 0.9317) (Figure 3(b)). The expression of ULK1 mRNA in GlycoA^+^ NRBC in the high-risk group (*n* = 14, 0.64 ± 0.91) was lower than that in controls (*n* = 23, 2.70 ± 3.27, *P* = 0.0275) and the low-risk group (*n* = 21, 4.98 ± 4.76, *P* = 0.0020). There was no significant difference between the controls and the low-risk group (*P* = 0.0695) (Figure 3(c)).

The mRNA expression of the autophagy suppressor gene—mTOR in GlycoA^+^ NRBC in high-risk group (*n* = 18, 2.81 ± 2.80)—was higher than that in the controls (*n* = 21, 1.29 ± 0.81, *P* = 0.0225) and the low-risk group (*n* = 25, 0.85 ± 0.74, *P* = 0.0135). There was no significant difference between the low-risk group and controls (*P* = 0.1109) (Figure 3(d)).

### 3.4. The Mitochondrial Dysfunction in GlycoA^+^ NRBC Measured in MDS Patients

**Figure 4:**
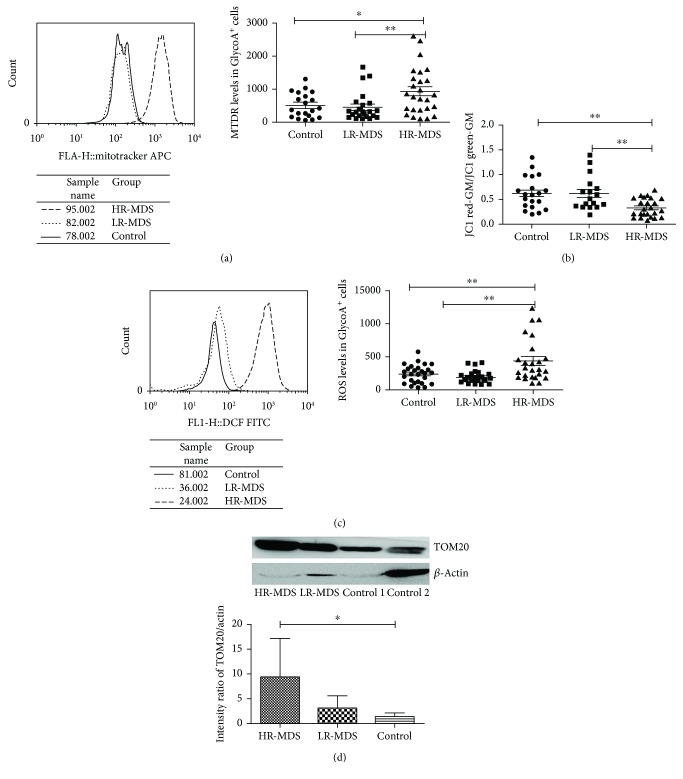
The mitochondrial dysfunction in GlycoA^+^ NRBC measured by FCM. (a) The amount of mitochondria (MTDR levels) in GlycoA^+^ NRBC in high-risk MDS was higher than that in controls and low-risk MDS. (b) The Δ*Ψ*_m_ in GlycoA^+^ NRBC in high-risk MDS was lower than that in controls and low-risk MDS. (c) The level of ROS in GlycoA^+^ NRBC in high-risk MDS was higher than that in controls and low-risk MDS. (d) The expression of the mitochondrial outer membrane protein TOM20 in GlycoA^+^ NRBC tested by Western blot. The intensity ratio of TMO20/*β*-actin tested by Western blot in the three groups (^∗^ *P* < 0.05, ^∗∗^ *P* < 0.01).

The amount of mitochondria (MTDR fluorescence levels) in GlycoA^+^ NRBC in the high-risk group (*n* = 26, 937.17 ± 707.85) was higher than that in controls (*n* = 20, 513.49 ± 372.33, *P* = 0.0194) and the low-risk group (*n* = 23, 461.74 ± 438.02, *P* = 0.0077). There was no significant difference between the controls and the low-risk group (*P* = 0.6811) (Figure 4(a)).

The levels of mitochondrial transmembrane potential (Δ*Ψ*_m_) in GlycoA^+^ NRBC in the high-risk group (*n* = 24, 0.33 ± 0.18) were lower than that in controls (*n* = 21, 0.61 ± 0.32, *P* = 0.0006) and the low-risk group (*n* = 17, 0.61 ± 0.34, *P* = 0.0014). There was no significant difference between the controls and the low-risk group (*P* = 0.9836) (Figure 4(b)).

The levels of ROS in GlycoA^+^ NRBC in the high-risk group (*n* = 25, 438.65 ± 322.83) were higher than that in controls (*n* = 27, 242.77 ± 136.87, *P* = 0.0057) and the low-risk group (*n* = 25, 197.40+ 95.07, *P* = 0.0008). There was no significant difference between the controls and the low-risk group (*P* = 0.1745) (Figure 4(c)).

The expression of the mitochondrial outer membrane protein TOM20 in GlycoA^+^ NRBC in the high-risk group (*n* = 3) was higher than that in controls (*n* = 3) (*P* = 0.0159). There was no significant difference between the high-risk group and the low-risk group (*n* = 3) (Figure 4(d)).

### 3.5. The Correlation Analysis with Altered Mitochondrial Function and Clinical Characteristics in MDS Patients

**Figure 5:**
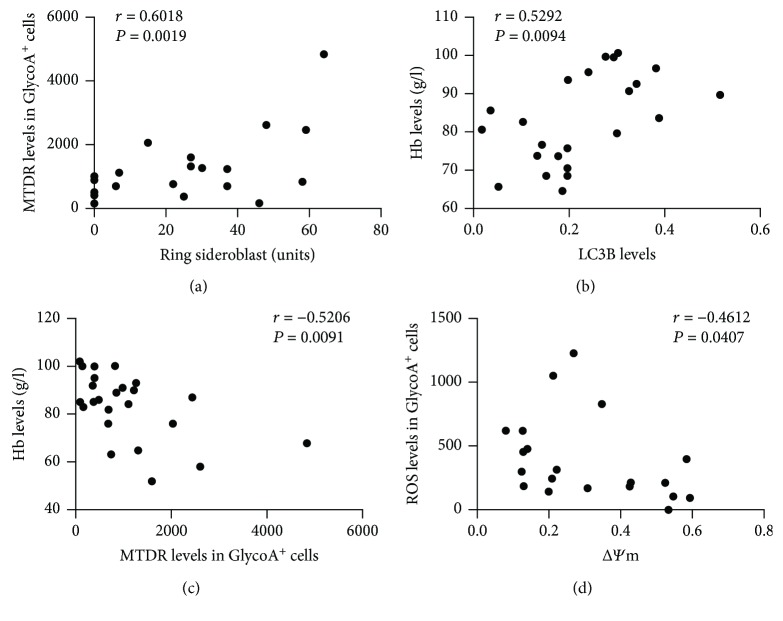
The correlation analysis between mitochondrial dysfunction in GlycoA^+^ NRBC and anemia associated with MDS. (a) In the high-risk MDS group, the amount of mitochondria in GlycoA^+^ NRBC was positively correlated with the count of ring sideroblasts in bone marrow samples (*P* < 0.01). (b) The level of LC3B in GlycoA^+^ NRBC was positively correlated with the hemoglobin level in high-risk MDS patients (*P* < 0.01). (c) The amount of mitochondria in GlycoA^+^ NRBC was negatively correlated with the hemoglobin level in high-risk MDS patients (*P* < 0.01). (d) The level of ROS in GlycoA^+^ NRBC was negatively correlated with Δ*Ψ*_m_ in high-risk MDS patients (*P* < 0.05).

In the high-risk MDS group, the amount of mitochondria in GlycoA^+^ NRBC positively correlated with the count of ring sideroblasts in bone marrow samples (*n* = 24, *r* = 0.6018, *P* = 0.0019) (Figure 5(a)). The level of LC3B in GlycoA^+^ NRBC positively correlated with the hemoglobin (Hb) levels in high-risk MDS patients (*n* = 23, *r* = 0.5292, *P* = 0.0094) (Figure 5(b)). The amount of mitochondria in GlycoA^+^ NRBC negatively correlated with the Hb level in high-risk MDS patients (*n* = 24, *r* = −0.5206, *P* = 0.0091) (Figure 5(c)). The ROS level in GlycoA^+^ NRBC had a negative correlation with Δ*Ψ*_m_ (*n* = 20, *r* = −0.4612, *P* = 0.0407) in high-risk MDS patients (Figure 5(d)).

## 4. Discussion

MDS are a group of clonal hematopoietic stem cell disorders characterized by cytopenia, dysplasia, and a high risk of transformation to acute myeloid leukemia (AML). The recent studies have reported that the loss of autophagy in murine hematopoietic stem/progenitor cells leads to bone marrow failure and development of age-related mitochondrial diseases such as MDS/AML [7, 13]. Autophagy, known as an adaptive response to stress, is an important way of orderly degradation and recycling of cellular components including mitochondria [14–16]. Mitophagy plays a key role in mitochondrial clearance during reticulocyte maturation [17–20]. Impaired mitophagy and mitochondrial dysfunction might be involved in ineffective hematopoiesis of MDS.

Mitophagy can be detected by the degradation of mitochondrial protein and autophagy markers such as LC3B [21]. Microtubule-associated protein-1 light chain-3 (LC3), present as a soluble form (LC3A) in the cytoplasm, is converted to a lipidated form (LC3B) which has been known to be an autophagosomal marker in autophagic activation [22, 23].

The BCL2-related protein NIX, which is upregulated during erythroid differentiation, is a mitochondrial outer membrane protein that acts as a mitochondrial receptor [24]. NIX gene knockout mice could acquire mild, nonlethal anemia [19]. The decreased NIX expression inhibits mitophagy by decreasing the ability of NIX to mediate autophagosomes. A study from Brazil determined the NIX expression of total bone marrow cells in MDS and AML patients [25]. The results showed that NIX was decreased in RAEB-1/RAEB-2 MDS patients compared to healthy donors. A significant reduction of NIX transcripts was also observed in AML with myelodysplasia-related changes (AML-MRC) and de novo AML compared with healthy donors. Moreover, lower NIX expression was an independent prognostic factor for worse overall survival (OS).

In our study, we analyzed the autophagosome and the change of mitophagy markers in erythroid precursors of MDS patients. The autophagy levels in NRBC were decreased in high-risk MDS patients compared with normal controls. The expressions of NIX and LC3B in NRBC were significantly lower in the high-risk MDS group compared with the low-risk MDS group and controls. It suggested that defective mitophagy occurred in erythroid precursors of MDS patients.

There are multiple protein kinases regulating autophagy, such as rapamycin complex 1 (mTORC1), AMP activated kinase (AMPK), and ULK autophagy proteins. mTORC1 acts as a major checkpoint regulating autophagy through the PI3K/Akt pathway [26] and AMPK [27, 28]. The mTOR and AMPK regulate autophagy through the inhibition of the phosphorylation of ULK1/2 [29]. ULK could phosphorylate and activate Beclin-1 [30]. The activation of ULK and Beclin-1 complexes could activate the downstream signaling of autophagy components [31–33]. A mild anemia was also found in cases where ULK1 is absent that led to a delay in the removal of mitochondria and ribosomes in erythroid cells. Abnormal activation of mTOR in erythrocytes could induce proteasome-mediated ULK1 degradation, which causes the defective clearance of mitochondria and erythrocyte macrocytosis [20]. Li-Harms et al. [13] reported the accumulation of mtDNA mutations in aged mtDNA mutant mice. Meanwhile, the abnormal activation of mTOR in erythrocytes led to anemia in mDNA mutant mice by inhibiting mitophagy in early erythroid progenitor cells and mitochondrial clearance in mature erythrocytes.

In our study, we tested the mRNA expression of the autophagy regulator gene in NRBC in MDS patients and controls. It showed that the mRNA expressions of AMPK and ULK1 in the high-risk MDS group were significantly lower than that in the low-risk MDS group and controls. The mTOR mRNA expression in the high-risk MDS group was significantly higher than that in the low-risk MDS group and controls. We inferred that increased mTOR in NRBC might suppress autophagy, facilitate abnormal mitochondrial accumulation, and finally lead to anemia in MDS. However, it is also possible that mitochondrial dysfunction could activate mTOR which inhibits the level of autophagy of NRBC.

After autophagy was damaged, mitochondria accumulated in various cells [34]. In the mice model to knockout the autophagy gene Atg7 in HSC, mitochondria accumulated in Atg7−/− erythrocytes with changed Δ*Ψ*_m_ causing cell death and anemia which was similar to the MDS phenotype [35]. TOM20 protein, a kind of mitochondrial outer membrane protein, is an essential receptor in mitochondrial protein import [36]. Increased TOM20 indicates that the number of mitochondria has increased and mitochondrial autophagy was defective.

In this study, we found that the amount of mitochondria in GlycoA^+^ NRBC increased in the high-risk MDS group, indicating the reduction of mitophagy in NRBC. TOM20 in the high-risk MDS group was also significantly higher than that of controls and the low-risk MDS group, which indicated that mitochondria accumulated in NRBC.

In addition, most cellular ROS (around 90%) are produced by mitochondria [37]. An increased number of mitochondria might accompany increased ROS production. However, increased ROS levels could damage both mitochondrial and nuclear genomes which would induce apoptosis [38, 39]. In the early stage of apoptosis, the mitochondrial membrane potential was decreased. There may be a direct or indirect crosstalk between autophagy and apoptosis [16, 40]. Raza et al. [41] analyzed apoptosis in bone marrow biopsy samples in 50 patients with MDS. Apoptosis was easily observed in erythroid progenitors and other bone-marrow-derived cells. They concluded that extensive intramedullary cell death might explain the paradox of pancytopenia despite hypercellular marrows in MDS patients. Other researchers reported that accumulated mitochondrial iron in MDS patients led to ineffective hematopoiesis [42].

In our study, we found that the high-risk MDS group had higher ROS levels and lower mitochondrial transmembrane potential in NRBC compared with the controls and the low-risk MDS group. Our results also showed that the amount of mitochondria in GlycoA^+^ NRBC positively correlated with the count of ring sideroblasts in bone marrow samples. Moreover, the ROS level negatively correlated with Δ*Ψ*_m_ in MDS patients. We hypothesized that damaged mitochondria which accumulated in the NRBC of MDS with increased ROS production might lead to early apoptosis and ineffective erythropoiesis.

In correlation analysis, our results showed that the LC3B level in NRBC had a positive correlation with the Hb level, and the amount of mitochondria in NRBC had a negative correlation with the Hb level in high-risk MDS patients. Herein, we inferred that impaired mitophagy in erythroid precursors might be related to the anemic pathogenesis in MDS patients with the increase of risk. Further studies need to be carried out.

A clinical trial reported that sirolimus, a kind of mTOR inhibitor, was effective in a subset of advanced MDS patients by activating autophagy [43]. In another study, azacitidine could promote both apoptosis and autophagy as a treatment for high-risk MDS patients. These results all indicated that targeting autophagy might have an activity in the treatment of MDS patients.

In summary, we found that NIX-mediated mitophagy was impaired in erythroid precursors in MDS patients, associated with an increased amount of damaged mitochondria and increased ROS level which might lead to early apoptosis and ineffective erythropoiesis. The increased amount of mitochondria in NRBC negatively correlated with anemia especially in high-risk MDS patients. These results indicated that mitophagy might involve the pathogenesis of anemia associated with MDS, and autophagy might be a novel target in treatment of MDS patients.

## Figures and Tables

**Table 1:** The characteristics of MDS patients.

Case	Sex/age	Diagnosis	Cytogenetics	IPSS
1	Male/63	RCMD	46, XY	Low
2	Male/58	RAEBII	46, XY	INT-2
3	Male/38	RAEBII	46, XY	INT-2
4	Female/70	RAEBII	46, XX	INT-2
5	Female/62	5q−	5q−	INT-1
6	Female/49	RARS	46, XX	INT-1
7	Female/79	RAEBII	46, XX	INT-2
8	Female/79	RAEBII	45, XX, −7	High
9	Female/29	RAEBII	20q−, 5q−, 7q−	High
10	Female/69	RAEBII	46, XX	INT-2
11	Male/30	RAEBII	47, XY, +8/46, XY	High
12	Male/50	RAEBI	46, XX	INT-1
13	Male/50	RCMD	47, XY, +8/46, XY	INT-1
14	Male/57	RAEBI	46, XY	INT-1
15	Female/74	RARS	46, XX	INT-1
16	Male/34	RARS	46, XY	Low
17	Male/42	RARS	46, XY, del20q11	INT-1
18	Female/47	RARS	46, XX	INT-1
19	Female/73	RAEBII	46, XX	INT-2
20	Female/64	RAEBI	46, XX	INT-1
21	Male/61	RAEBII	46, XY	INT-2
22	Male/59	RAEBI	46, XY	INT-1
23	Male/59	RAEBII	46, XY	INT-2
24	Male/59	RAEBII	46, XY	INT-2
25	Male/68	RAEBII	46, XY, +8/45, XY+8, −6, −7	High
26	Male/76	RAEBII	No result	INT-2
27	Male/52	RN	No result	Low
28	Female/64	RAEBII	46, XX	INT-2
29	Female/57	RAEBII	46, XX	INT-2
30	Male/62	RA	46, XY	INT-1
31	Female/74	RCMD	46, XX	INT-1
32	Male/65	RCMD	46, XY, del17q31	INT-2
33	Female/76	RAEBII	del5q33, del5q31, del7q311, del7q3	High
34	Female/59	RAEBI	46, XY, 13q+	INT-2
35	Female/51	RAEBI	46, XX	INT-1
36	Male/62	RA	46, XY, 13q+	INT-1
37	Female/61	RCMD	46, XX	INT-1
38	Male/46	RCMD	46, XY, −2, −12, +mar, 19+, 9P+	INT-2
39	Female/77	RAEBII	45, XX, −5, −2, 45, XX, +mar, −5, 3P−	High
40	Male/70	RAEBI	46, XY	INT-1
41	Male/46	RAEBII	45, XY, −7, −21, +mar(21q+)/45, XY, −7/46, XY, −21, +mar21q+	High
42	Male/27	RAEBII	3p+, −18, +mar	High
43	Male/60	RAEBII	45, XY, −7	High
44	Female/67	RCMD	46, XX	INT-1
45	Male/61	RCMD	45-46, XY, 21p+	INT-1
46	Female/68	RCMD	46, XX	INT-1
47	Male/71	RCMD	46, XY	INT-1
48	Male/62	RAEBII	46, XY	INT-2
49	Male/67	RAEBII	46, XY	INT-2
50	Female/56	RCMD	17P+, +8	INT-1
51	Female/60	RCMD	46, XX	INT-1
52	Female/46	RCMD	46, XX	INT-1
53	Male/58	RCMD	46, XY	INT-1
54	Male/48	RCMD	46, XY	INT-1

## Supplementary Materials

Supplementary Materials Supplementary Figure 1: the LC3 expression in GlycoA^+^ NRBC from MDS and controls treated with rapamycin and 3-methyladenine (3-MA).
