# Supplementary figures and images for: Impaired Mitophagy of Nucleated Erythroid Cells Leads to Anemia in Patients with Myelodysplastic Syndromes
Source: Oxid Med Cell Longev. 2018 Jun 3;2018:6328051. doi: 10.1155/2018/6328051 (PMC6008680; doi:10.1155/2018/6328051)

Supplementary figure 1 The LC3 expression in GlycoA+ NRBC from MDS (n=3) and controls (n=2).

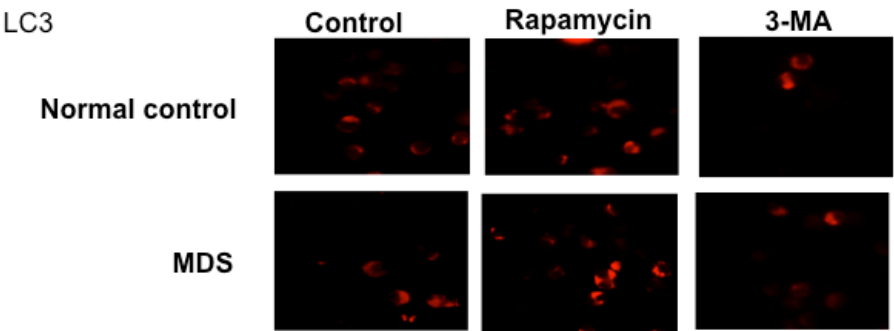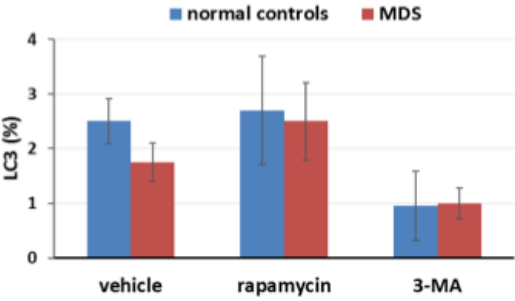

Supplement: Supplementary Materials — Supplementary Figure 1: the LC3 expression in GlycoA+ NRBC from MDS and controls treated with rapamycin and 3-methyladenine (3-MA). [file 6328051.f1.pdf]
